# Clones reactive to apoptotic cells and specific chemical adducts are prevalent among human thymic B cells

**DOI:** 10.3389/fimmu.2024.1462126

**Published:** 2024-10-21

**Authors:** Andrea Hertel, Talita Aguiar, Shunya Mashiko, Sarah Núñez, Carolina Moore, Baoshan Gao, Mattea Ausmeier, Poloumi Roy, Emmanuel Zorn

**Affiliations:** ^1^ Columbia Center for Translational Immunology, Department of Medicine, Columbia University Irving Medical Center, New York, NY, United States; ^2^ Medical Department IV - Großhadern, LMU University Hospital, Ludwig-Maximilians-Universität München, Munich, Germany; ^3^ Facultad de Medicina y Ciencia, Universidad San Sebastián, Santiago, Chile; ^4^ Centro Ciencia y Vida, Santiago, Chile; ^5^ Transplant Center, Department of Surgery, Massachusetts General Hospital, Harvard Medical School, Boston, MA, United States; ^6^ Institute of Anatomy and Cell Biology, Faculty of Medicine, Martin-Luther-University Halle-Wittenberg, Halle, Germany

**Keywords:** thymic B cells, chemical adducts, natural antibodies, apoptotic cells, polyreactivity

## Abstract

**Introduction:**

Thymus resident B cells were described more than 40 years ago. In early human life, these cells are found predominantly in the medulla and overwhelmingly display an unswitched IgM+ phenotype. The reactivity of thymic IgM B cells, however, is still unclear.

**Methods:**

Here, we generated 120 IgM-producing B cell clones from 3 separate thymus specimens obtained from infant, adolescent, and adult donors. Using flow cytometry and a unique high-dimensional ELISA platform, we investigated the clones’ reactivity to apoptotic cells as well as to common chemical adducts exposed on modified amino acids and other macromolecules.

**Results:**

Regardless of the age, approximately 30-40% of thymic IgM B cells reacted to apoptotic cells. Further, 30-40% displayed reactivity to at least one adduct, including malondialdehyde, Homocysteine, and NEDD 8. Four distinct reactivity patterns were identified through this profiling. Notably, a significant association was observed between reactivity to apoptotic cells, and to one or more adducts, suggesting that the same determinants were recognized in both assays. Additionally, thymic IgM B cells reactive to adducts were more likely to recognize intra-nuclear or intra-cytoplasmic structures in Hep-2 cells as revealed by immunofluorescence staining.

**Conclusion/Discussion:**

Collectively, our findings suggest that thymic IgM B cells actively uptake apoptotic bodies and cellular debris in the medulla by binding specific chemical adducts. This mechanism could underpin their antigen-presenting function and further support their role in T-cell negative selection.

## Introduction

The thymus is a primary lymphoid organ known for its role in the differentiation of bone marrow (BM)-derived progenitor T-cells and the induction of central tolerance, a process that eliminates self-reactive T cells during their development (1). A sizeable contingent of B cells is also found in the thymus, where it occupies approximately 30% of the medullary space (2–4). A number of studies have characterized these thymic B cells in mice and humans. Our own group revealed that human thymic B cell subsets change across the lifespan (3). While these cells are predominantly IgM+ in early life, the frequency of class-switched IgG+ and IgA+ thymic B cells increase with age. Moreover, aside from the medulla, the thymic perivascular space was identified as a functional niche for plasma cells, including high-frequency clones reactive to common viruses (3). In a separate study, Cordero et al. showed the differentiation of plasma cells directly in the thymus of neonates and infants (5).

Regarding the dominant population of medullary B cells, several groups using mouse models investigated their potential role in the differentiation of thymocytes. These studies provided supportive evidence that these cells contribute to negative selection of autoreactive T cells (6–9). Notably, it was shown that a large proportion of thymic B cells react to generic self-antigens, suggesting a link between their specificity to self-antigens and their role in the deletion of autoreactive clones (8, 9). This function requires a unique licensing process of B cells expressing AIRE (9). Expression of this essential transcription factor correlated with the upregulation of MHC II and CD80. Licensing requires CD40 signaling through direct interaction with CD4+ thymocytes and enables thymic B cells to present self-antigens to thymocytes, contributing to the negative selection of autoreactive T cells and assisting AIRE-expressing medullary stromal cells (mTECs), traditionally considered as the main cell type involved in T cell negative selection (9). In contrast to their mouse counterparts, human thymic B cells remain largely unexplored, warranting further investigations.

Earlier studies from our group reported that the majority of the thymic B cells in human newborns and infants express IgM (3). Here, we aimed to interrogate the reactivity of these B cells, especially toward apoptotic cells as a distinctive feature of natural antibodies (Nabs) (10, 11). Furthermore, we previously observed that Nabs often bind to apoptotic cells by recognizing specific chemical moieties such as malondialdehyde (MDA), or other chemical adducts (12). We thus ought to examine the specificity of thymic IgM B cells to common chemical adducts and possibly identify consensus determinants. To achieve these objectives, we used a unique platform to detect the reactivity to 93 chemical adducts. With this platform, we assessed the reactivity profiles of monoclonal IgM secreted by 120 B cell clones derived from 3 thymus specimens.

## Materials and methods

### Human subjects and specimens

Human Thymus tissue samples were obtained from three subjects, aged 5 weeks, 15 years and 39 years. The pediatric donors (5 weeks, 15 years) were patients diagnosed with congenital heart disease who underwent corrective cardiac surgery at Boston Children`s Hospital. The adult donor receives a left-ventricular assist device at Massachusetts General Hospital (MGH). Buffy coats from 4 healthy donors were obtained from the New York Blood Center. This study was approved by the Institutional Review boards of both MGH and Columbia University. All samples were de-identified according to the Protected Health Information regulations.

### Sample processing

Thymic specimens were processed immediately after collection. The tissue was washed extensively with phosphate-buffered saline (PBS) to remove blood before being homogenized using a gentleMACS tissue dissociator (Miltenyi Biotec) and filtered through a 40 μm cell strainer (BD Biosciences). Thymocyte cell suspensions were then cryopreserved in heat-inactivated fetal bovine serum (FBS) (Atlanta Biologicals) supplemented with 10% dimethyl sulfoxide (DMSO) and stored in liquid nitrogen.

### Isolation and immortalization of B cell clones

The procedure for isolating and immortalizing B cell clones has been described previously (13). In brief, Epstein Barr virus-transformed B cell clones were generated by incubating B cells immunopurified using anti-CD19 microbeads (Miltenyi Biotech, Auburn, CA) with supernatant from Epstein-Barr-virus producing B95-8 marmoset cells in the presence of the Toll-like receptor 9 agonist CpG 2006 at a concentration of 2.5 μg/ml (InvivoGen, San Diego, CA). Transformed clones were generated by limiting dilution in 96- well plates on a layer of irradiated THP-1 feeder cells. Cells were seeded at 0.3 cell/well in 96 well plates. Less than 30 growing clones/plate ensuring monoclonality. The established clones were maintained in Roswell Park Memorial Institute 1640 medium (RPMI) supplemented with 10% heat-inactivated fetal bovine serum, 4 mM glutamine, 1 mM sodium pyruvate, and 10 mM Hepes, and antibiotics. Cells were maintained for up to 3 months at a density of 0.5-1.5 x 10^6^ cells/ml. Cell culture supernatant was collected on a weekly basis and stored at -80°C. The presence of IgM, IgG and IgA secreted in the culture supernatant by the individual B cell clones was assessed by ELISA (ThermoFisher, Waltham, MA).

### Flow cytometry

Flow cytometry was utilized to assess the reactivity of IgM secreted by immortalized thymic B cell clones toward apoptotic Jurkat T-cells. Apoptosis was induced in Jurkat cells through ultraviolet irradiation (240 x 10-3J) using a UV stratalinker 2400 (Stratagene, Santa Clara, CA) and incubated for 18 hours. A 1:1 mixture of viable and apoptotic cells (1 x 10^6^ cells) was incubated for 30 minutes at 37°C with 100 μl of IgM-containing cell culture supernatants of immortalized B cell clones. Cells were then washed in PBS and incubated with FITC-conjugated anti-human IgM F(ab´)_2_ (Jackson ImmunoResearch Labs, West Grove, PA). Before the acquisition, samples were co-stained with 7-AAD Viability and allophycocyanin-conjugated Annexin V (Biolegend, San Diego, CA) according to the manufacturer’s instructions. Cells were then acquired using an Accuri C6 flow cytometer (BD Biosciences, San Jose, CA) after gating on apoptotic cells. All samples were assessed using the same experimental settings to minimize inter-experiment variations.

### Adduct panel

The ELISA panel used in these studies is a modified version of the panel described in a previous report (12). The panel comprises 93 adducts and controls, including (i) forty-six synthetic 5-mer peptides, each featuring a modified amino acid flanked by four unmodified arginine residues, two in each side (5-mer peptides with matching unmodified residues were included into the panel as controls); (ii) 6 modified amino acids; (iii) eight post-translational modification (PTM) compounds such as small ubiquitin-related modifier (SUMO) and ubiquitin; and (iv) thirty-three cofactors, coenzymes, and metabolites. Additionally, the panel includes malondialdehyde-modified bovine serum albumin (MDA-BSA) and MDA-lysine/arginine peptides. These MDA-modified protein/peptides were prepared by incubating acid-hydrolyzed 1,1,3,3-tetramethoxypropane (Sigma-Aldrich, St. Louis, MO, USA) with BSA or lysine/arginine peptides. Specifically, 2 M 1,1,3,3-tetramethoxypropane was hydrolyzed in 96mM HCl for 15 min at 37°C followed by neutralization with NaOH. BSA (2 mg/ml) or lysine/arginine peptides (10 mg/ml) were then incubated with 0.2 M MDA for 3 hours at 37°C. Extensive dialysis against 1× PBS was conducted at 4°C for 36 hours to remove any residual substances. All in the panel included adducts and controls are displayed in **Supplementary Table 1**, including their sources and used (working) concentrations.

### Detection of adduct-reactive antibodies

Supernatant IgM reactivity to 93 chemical adducts was assessed using a previously described ELISA platform (12). Briefly, CorningTM Clear Polystyrene 96-Well EIA/RIA Microplates (Corning Incorporated) were coated in duplicates with the specific adducts, controls and compounds and incubated at 4°C for 20 hours. Synthetic peptides with modified amino acids, large PTM compounds, and small compounds were coated at 10μM, 1μM, and 1mM, respectively, as detailed in **Supplementary Table 1**. ELISA plates were washed twice with PBS Tween (PBST) and blocked with 3% BSA (Fisher Scientific Inc.) in PBS for two hours at 37°C. IgM in cell culture supernatants were quantified using a Human IgM ELISA kit (IHUIGMKT, Innovative Research) and then used at a concentration of 25 ng/ml in PBST and added to the plates. After three hours of incubation at room temperature, plates were washed five times with PBST. Subsequently, plates were incubated at room temperature with horseradish peroxidase (HRP)–conjugated anti-human IgM affinity-purified F(ab′) _2_ fragment secondary antibody (Donkey IgG; Jackson ImmunoResearch Laboratories Inc., West Grove, PA) diluted 1:4000 in blocking buffer. Following another five washes with PBST, HRP activity was revealed with 3,3′,5,5′-tetramethylbenzidine (TMB; Fisher Scientific Inc.). The enzymatic reaction was stopped after five minutes with H2SO4 (1N). Reactivity to adducts was measured as the optimal density (OD). Validation ELISAs were performed for individual adducts following a similar procedure. Plates were coated with the adducts to be validated or respective controls. In addition to positive samples, negative control samples were included for each validation experiment. Positivity was defined as values above the median plus standard deviation of the corresponding unmodified control peptide as described in our previous report (12).

### Immunofluorescence assays

Human epithelial cell line (Hep-2)-cell coated slides (AN-1012, Bion Enterprise Ltd, Des Plaines, IL) were incubated at room temperature with 25 μl of undiluted clone supernatants for 30 minutes. Slides were then washed with PBS for 10 minutes and incubated at room temperature for 30 minutes with fluorescein isothiocyanate-conjugated goat anti-human Ig (CCP-9970, Bion Enterprise Ltd, Des Plaines, IL). After another wash with PBS, immunofluorescence was then visualized using a Zeiss LSM 900 confocal microscope at 20x-magnification. Images were acquired using a Plan-Apochromat 20x/0.8 M27 objective, with FITC filter set (excitation: 495 nm, emission: 519 nm). For each image, an average of at least 8 scans was performed to reduce noise. For Hep-2 assays, clones showing staining intensity markedly above that of the negative control were considered positive.

Immunofluorescence staining was performed on 5- μm- thick formalin-fixed paraffin-embedded (FFPE) tissue sections. Sections were deparaffinized with xylene and rehydrated in sequential steps with solutions containing decreasing concentrations of ethanol (100%, 95% and 70%). A DIVA Decloaker (BIOCARE MEDICAL) was used to perform antigen retrieval. Background staining was blocked with 15 μg/ml AffiniPure F(ab’)2 Fragment Goat Anti-Human IgM, Fc5µ Fragment Specific (Jackson ImmunoResearch, 109-006-129) diluted in 1% BSA (Fisher Scientific Inc.) for 24 h at 4°C. Slides were subsequently incubated overnight at 4°C with monoclonal cell culture supernatant, diluted 1:5 in 3% BSA, followed by 3 washes with PBS 1x. Slides were then incubated for 1 hour at room temperature with a goat anti-Human IgM (Heavy chain) Cross-Adsorbed Secondary Antibody, conjugated to Alexa Fluor^®^ 647 (Invitrogen, A-21249) (5μg/ml), followed again by 3 washes with PBS 1x. Lastly, slides were mounted with Vectashield Vibrance^®^ Antifade Mounting Medium with DAPI (H-1800) (Vector laboratories). Images were acquired with a Zeiss LSM 900 confocal microscope at 10X and 63X magnification using EC Plan-Neofluar 10x/0.30 M27 and Plan-Apochromat 63x/1.40 Oil DIC M27 objective. Two channels were used to visualize Alexa Fluor 647 (excitation: 653 nm, emission: 668) and DAPI (excitation: 353nm, emission: 465nm). For each image, an average of 16 scans was performed to reduce noise.

Further Image processing and analysis for all images were performed using ZEN Blue 3.4 software (Carl Zeiss Microscopy GmbH). All pictures acquired in the same set of experiments were analyzed using the same settings for consistency and processed based on the negative control.

### Statistical analysis

2x2 Fisher Exact test was performed (** P < 0.001 and ****P < 0.0001) to compare IgM reactivity to apoptotic cells between different age groups, to compare anti-adduct IgM reactivity between the different age groups, and to compare the reactivity to apoptotic cells obtained by Flow cytometry with anti-adduct IgM reactivity obtained by ELISA. Data analysis was conducted using GraphPadPrism 10. Standardization and unsupervised clustering of OD values obtained by ELISA were conducted using R. version 4.3.1. All used packages are listed in **Supplementary Table 2**.

## Results

### Thymic IgM B cell reactivity to apoptotic cells

**Figure 1:**
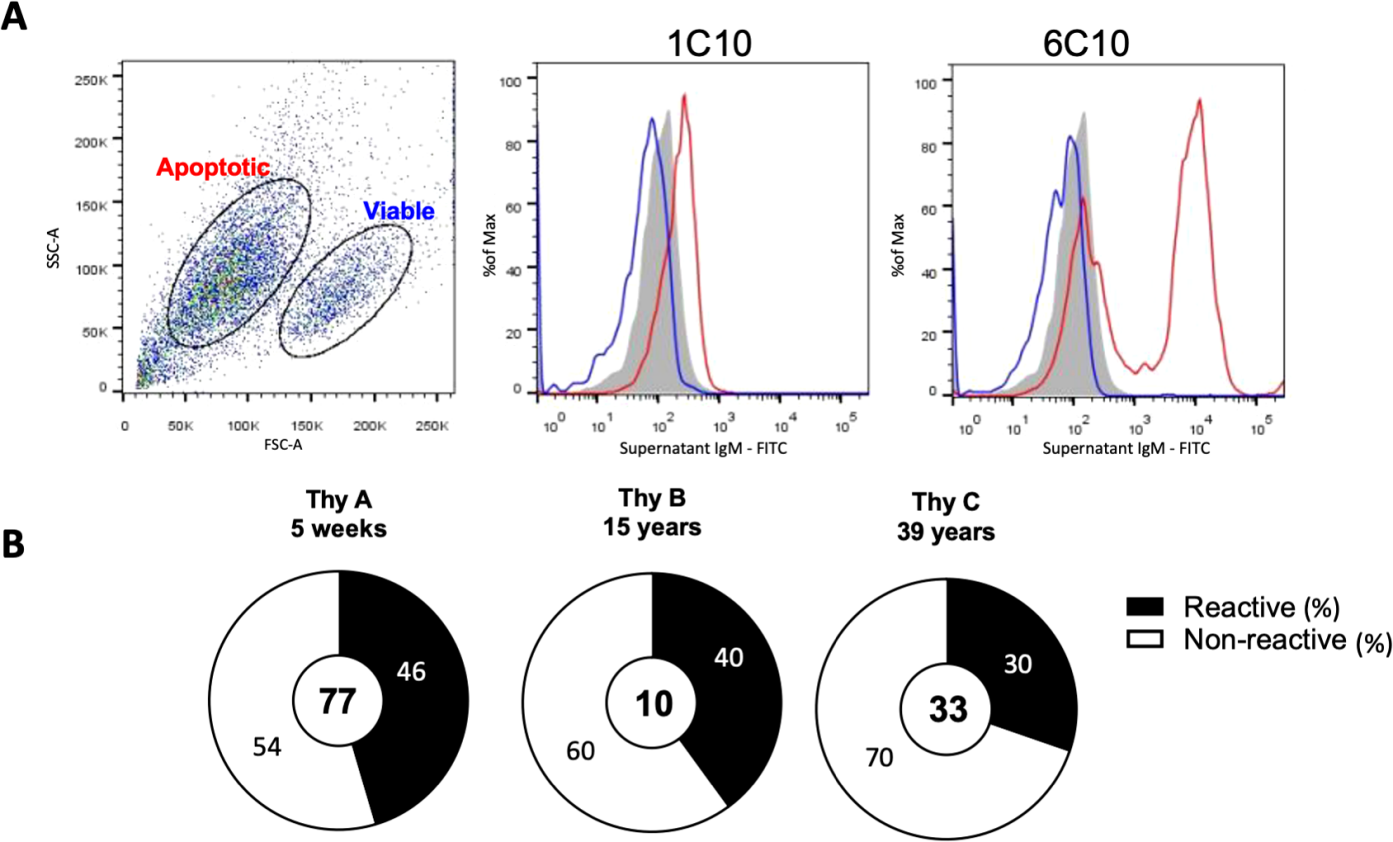
Thymic B cell IgM reactivity to apoptotic cells. **(A)** Reactivity of IgM produced by immortalized thymic B cell clones to apoptotic Jurkat T-cells as revealed by flow cytometry. Representative plots showing a non-reactive clone (1C10) and a reactive clone (6C10) are depicted. Grey histograms show baseline staining with the secondary antibody alone after gating on apoptotic cells. Blue histograms show staining on viable cells and red histograms show staining on apoptotic cells. **(B)** Frequency of B cell clones reactive in the three thymus specimens. The number of clones tested is indicated in the center of each doughnut. P-values were calculated using the 2 x 2 Fisher exact test.

To characterize their reactivity profile, we immortalized thymic B cell clones derived from three donors aged 5 weeks (Thy A), 15 years (Thy B), and 39 years (Thy C) who underwent cardiac surgery. A total of 178 CD19^+^ B cell clones were established from the three donors (Thy A n=88, Thy B n= 41, Thy C n= 48). **Supplementary Figure 1** reports the secretion of IgM, IgG and IgA among all thymic B cells. In this study we focused exclusively on IgM-secreting thymic B cells (Thy A n=77, Thy B n= 10, Thy C n= 33). We first assessed the IgM clones’ reactivity to apoptotic cells by flow cytometry using the monoclonal IgM secreted in the culture supernatant as previously described (14). Most thymic B cells displayed an unequivocal negative or positive reactivity pattern toward apoptotic cells as illustrated in **Figure 1A** and **Supplementary Figure 2** A for representative negative and positive clones. Results for all clones are reported in **Supplementary Figures 2B–D**. Overall, the proportion of thymic B cells reactive to apoptotic cells was very consistent for the three donors irrespective of their age: 46% for the 5-week-old (Thy A), 40% for the 15-year-old (Thy B) and 30% for the 39-old-year thymus (Thy C) as shown in **Figure 1B**. For comparison we also assessed the baseline frequency of IgM B cells reactive to apoptotic cells in the peripheral blood of 4 healthy subjects using the same techniques as described in the methods section. As depicted in **Supplementary Figure 3** the frequency ranged from 2.3 to 5.8% of all immortalized IgM+ clones (n=1290), far less than the frequency observed among thymic B cells.

### Thymic IgM B cell reactivity to chemical adducts and its association to anti-apoptotic cell reactivity

**Figure 2:**
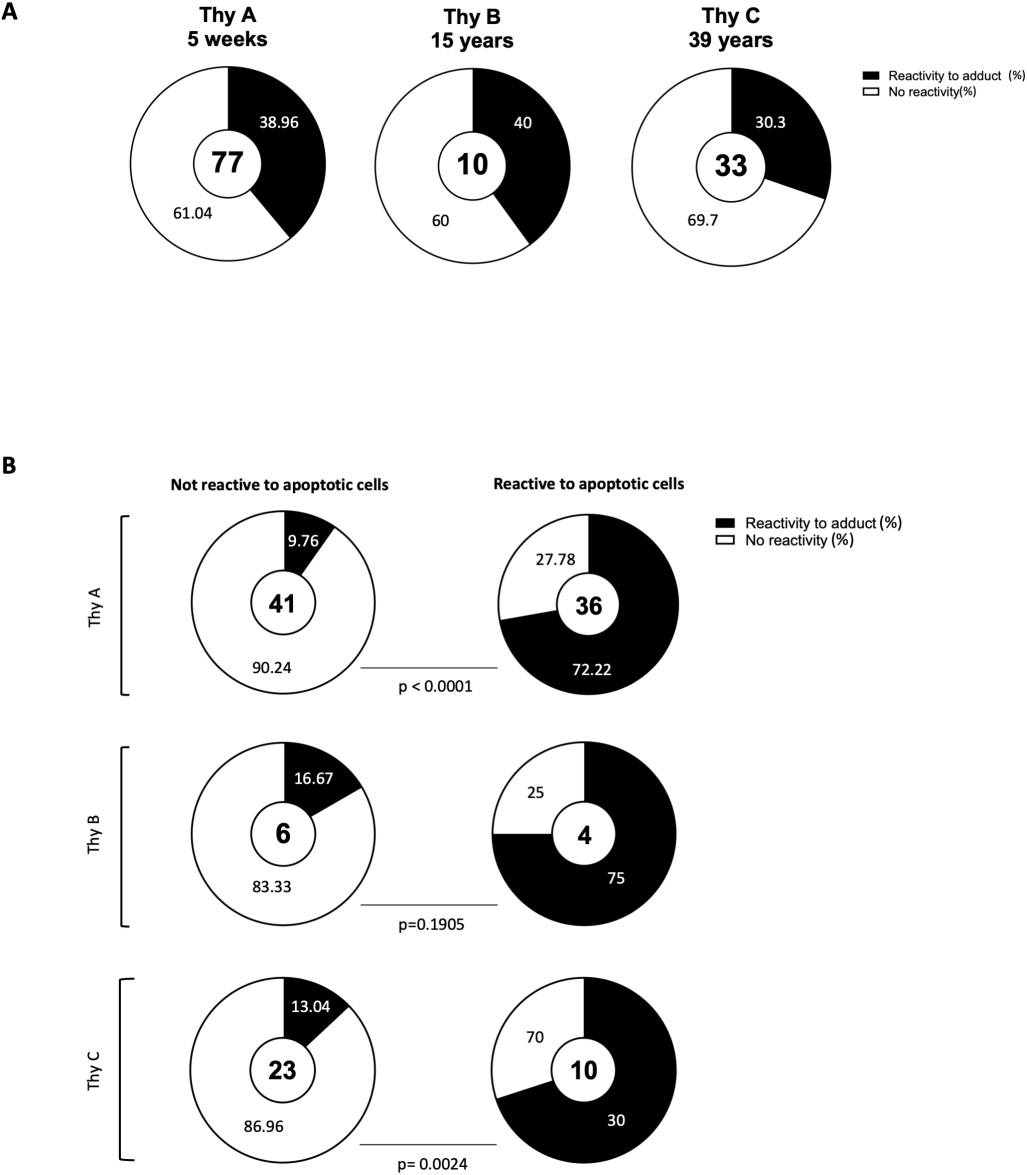
Association between reactivity to apoptotic cells and to chemical adducts. **(A)** Proportion of thymic IgM B cell clones reactive to at least one out of the 93 adducts assessed by multidimensional ELISA for the three thymus specimens. **(B)** Proportion of thymic IgM B cell clones reactive to at least one adduct among clones reactive or not-reactive to apoptotic cells The number of total clones assessed is indicated in center of each donut chart. P-values were calculated using the 2 x 2 Fisher exact test.

**Figure 3:**
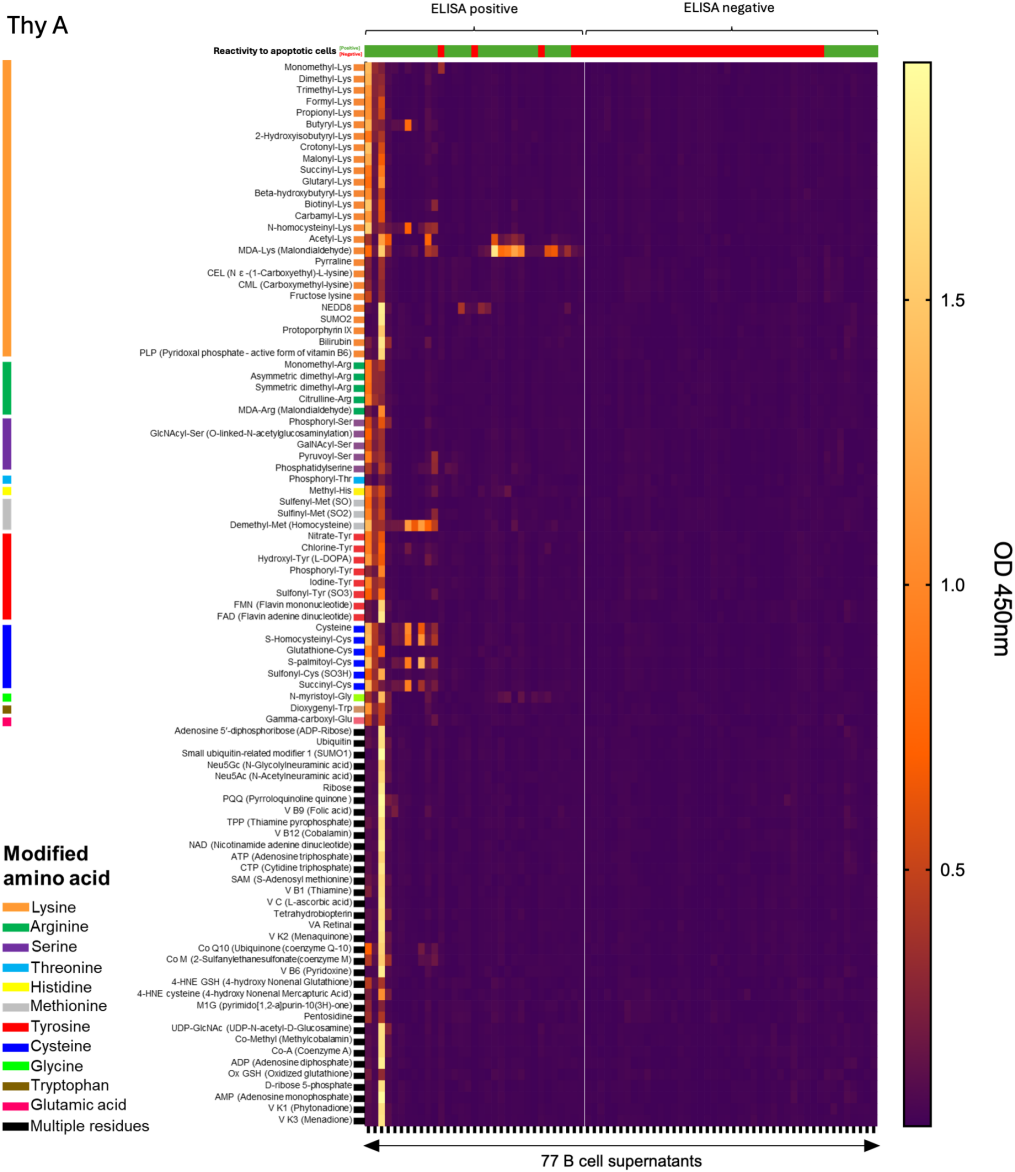
Thymic B cell IgM reactivity to chemical adducts. Reactivity of IgM mAb secreted by 77 thymic IgM B cell clones generated from a 5-week-old thymus (Thy A) to 93 chemical adducts using a heatmap representation. Adducts are grouped by modified amino acids. mAb were categorized as reactive to at least one adduct (left) or nonreactive (right).

To further examine the clones’ profiles, we used a multi-dimensional ELISA to detect IgM reactivity to 93 common chemical adducts (**Supplementary Table 1**). The panel included PTMs on 11 amino acids, oxidation-related modification as MDA, advanced glycation end products (e.g., pyrraline), and certain co-enzymes (e.g., flavin adenine dinucleotide, FAD). Individual profiles were obtained for all 120 monoclonal IgM. As reported in **Figure 2A**, 30.3% to 40% of thymic IgM B cell clones were reactive to at least one adduct. No significant difference was observed between infant, child, and adult donors. A 2x2 Fisher test revealed a highly significant association between reactivity to adduct and to apoptotic cells for Thy A (p<0.0001) and Thy C (p=0.0024). While **Figure 2B** shows the same trend, the association did not reach significance for Thy B (p=0.1905), mostly due to a limited sample size. The detailed reactivity patterns of the 77 Thy A clones are depicted in **Figure 3**. Those obtained for Thy B and Thy C clones are reported in **Supplementary Figure 4**. As apparent in these figures, clones could be categorized as reactive to at least one adduct (left part of the heatmaps) or not reactive to any adduct included in our panel (right part of the heatmaps). Reactivity to individual chemical adducts observed during the screening was confirmed by ELISA for all positive clones except for 1 clone initially binding to monomethylated lysine and 2 clones initially reactive to phosphatidylserine as illustrated in **Supplementary Figure 5**.

### Identification of distinct thymic IgM B cell reactivity profiles

**Figure 4:**
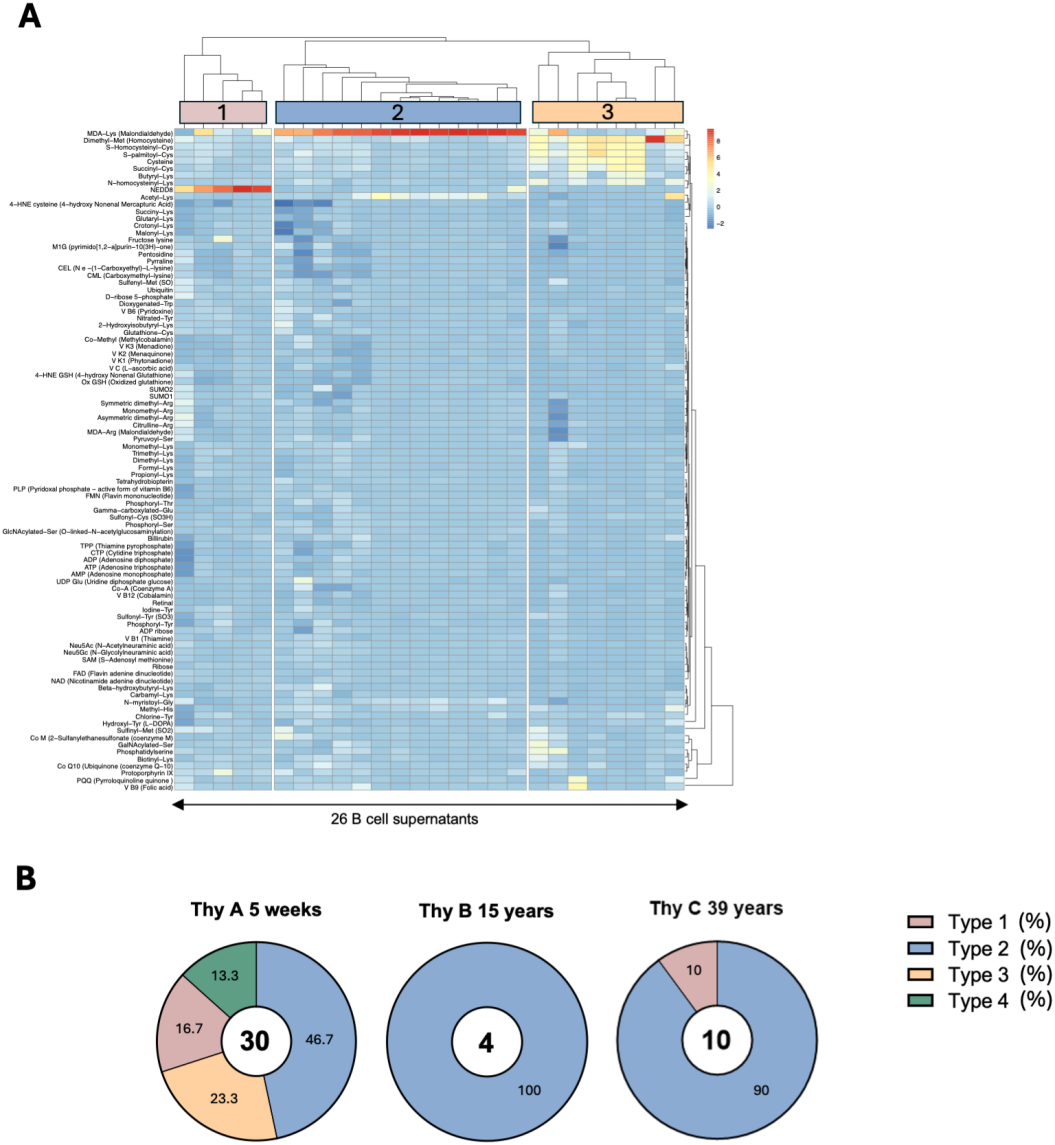
Characterization of thymic IgM B cell clone reactivity profiles. **(A)** Unsupervised hierarchical clustering of thymic IgM B cell clone reactivity to adducts, among adduct-reactive clones generated from a 5-week-old thymus (Thy A) after excluding 4 polyreactive clones. **(B)** Distribution of reactivity types for the three thymus specimens.

To identify specific reactivity patterns among Thy A IgM clones, we conducted an unsupervised hierarchical clustering analysis of standardized signal values obtained through the screening, which is depicted in **Figure 4A**. Of note, this analysis excluded 4 clones displaying a clearly polyreactive reactivity profile as shown in the left part of the heatmap in **Figure 2**. Results unveiled three distinct reactivity patterns consistent with the visual interpretation of the heatmap shown in **Supplementary Figure 6** after normalization via z-score transformation. Based on these reactivity profiles, the 3 types of clones identified through this analysis can be described as follows: Type 1, thymic B cells exhibiting prominent reactivity to NEDD8; Type 2 clones showing reactivity mostly to MDA either affixed to lysine or arginine. Type 3 B cells primarily reacting to demethylated methionine (Homocysteine) but also cross-reacting to N-homocysteinyl-lysine, cysteine, S-homocysteinyl-cysteine, and S-palmitoylated-cysteine. Type 4 corresponded to the polyreactive samples that were set aside from the clustering analysis. In total, 4 types of IgM B cells were characterized in Thy A. For Thy B, all positive clones aligned with the Type 2 reactivity profile whereas for Thy C clones fitting the Type 1 and Type 2 reactivity profiles were observed as illustrated in **Supplementary Figure 4**. A summary of the clone type distribution among the 3 thymus specimens is provided in **Figure 4B**, underscoring the predominance of Type 2 IgM B cells across different ages. Additionally, **Supplementary Figure 7** shows the distribution of anti-apoptotic cell reactivity for the different profiles. The rate of anti-apoptotic clones among adduct-reactive clones was 74-100%.

### Immunofluorescence assays for self-reactivity

**Figure 5:**
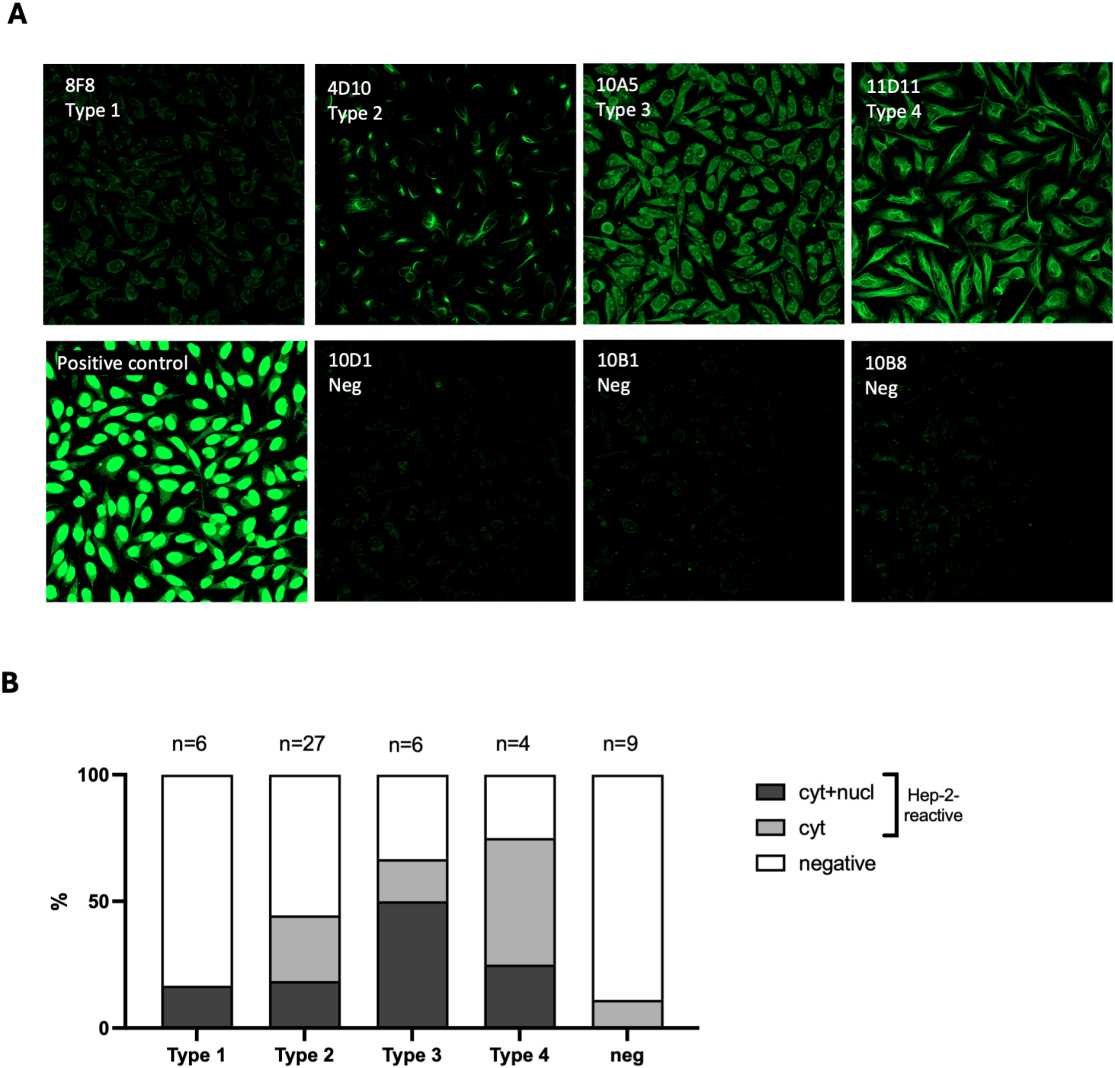
Anti-Adduct IgM reactivity to Hep-2 cells. **(A)** Staining pattern of representative thymic IgM B cell clones reactive to Hep-2 cells. Each clone corresponds to a distinct reactivity type. Clones 10D1, 10B1, and 10B8 are non-reactive negative controls. **(B)** Distribution of clones with distinct Hep-2 reactivity patterns (cytoplasmic, nuclear, and negative) among thymic IgM B cell clones reactive to adducts according to their types (1–4).

The HEp-2, indirect immunofluorescence assay (IFA) is a widely used method to detect self-reactive antibodies e.g., antinuclear antibodies (ANAs), in the serum of patients with autoimmune diseases (15, 16). This assay often reveals distinctive reactivity patterns that have been associated with different autoimmune conditions (16). We used the HEp-2 assay to examine the staining patterns of the 4 thymic IgM B cell types identified above. IFA was conducted with a representative set of 52 clonal IgM from the 3 donors. Three main staining patterns were observed: either no staining, cytoplasmic staining or a combination of nuclear/nucleolar and cytoplasmic staining consistent with ANAs and often being associated with SLE or rheumatoid arthritis (17). A representative sample of these staining patterns and their distribution by clone types are presented in **Figure 5**. As shown in this figure, reactivity to chemical adducts was accompanied by Hep-2 staining for most clones. More specifically, 16.6% of Type 1 clones exhibited reactivity toward both nuclear and cytoplasmic antigens. In the Type 2 group, 18.5% displayed nuclear and cytoplasmic staining, and an additional 26% only reacted to cytoplasmic structures. The Type 3 clones had the highest proportion of ANAs, with 50% showing the characteristic nuclear and cytoplasmic staining pattern, while 16.6% bound only to cytoplasmic antigens. Polyreactive Type 4 clones were the most reactive to HEp-2, with 75% of them displaying some form of staining. In sharp contrast, negative clones, which did not bind to any adducts, showed the lowest reactivity to HEp-2. Only 1 out of 9 clones tested bound to cytoplasmic antigens.

### Anti-adduct IgM derived from thymic B cells react to thymic medullary cells

**Figure 6:**
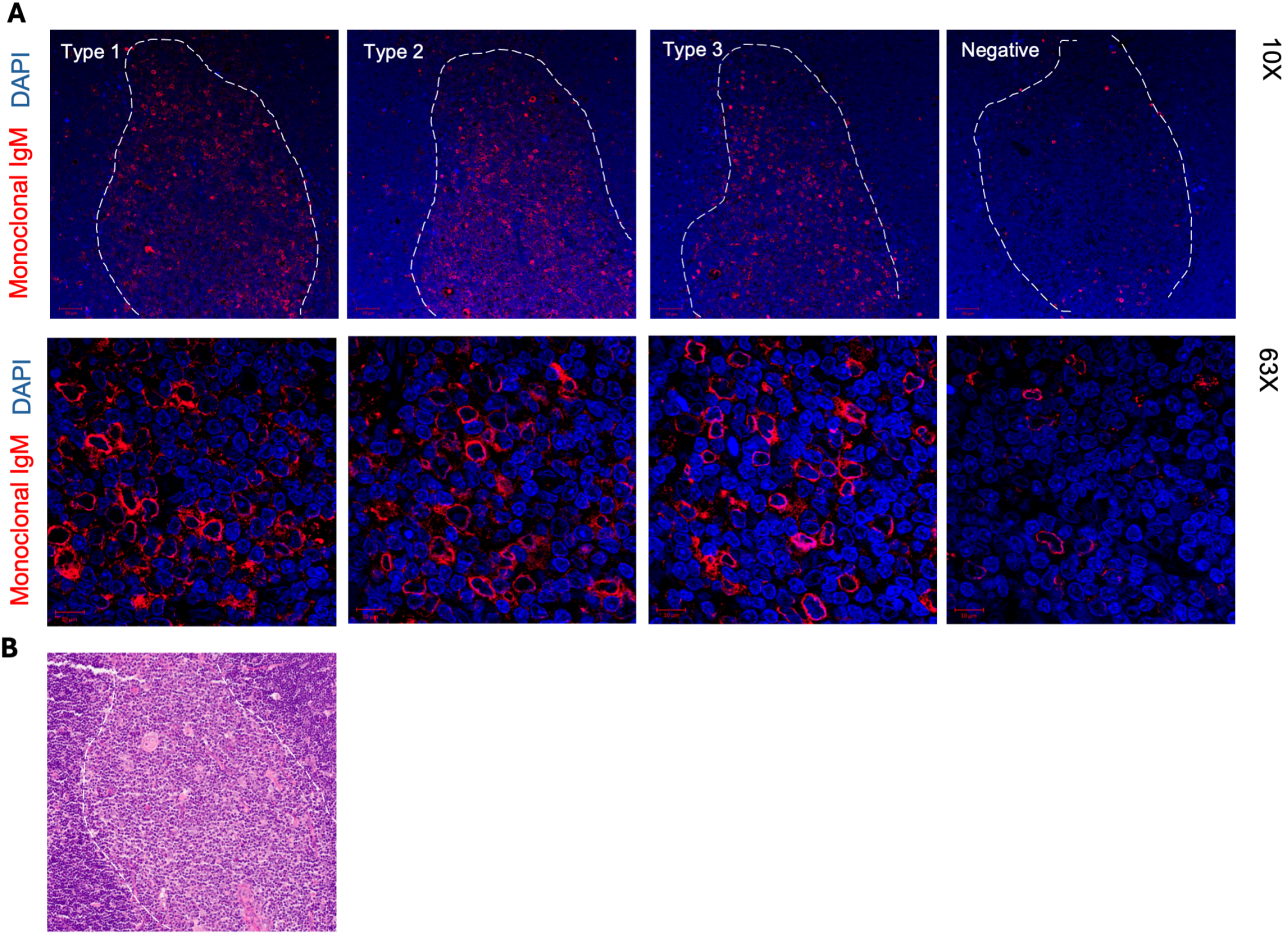
Immunofluorescence staining of thymic tissue with IgM mAB secreted by of thymic IgM B cell clones. **(A)** FFPE tissue sections of a 15-week-old thymus (Thy A) were stained with IgM mAb from thymic B cell clones with different adduct reactivity type. The medulla is delineated with a white dotted line. Higher magnification images shown in lower panels depict staining in the medulla. **(B)** Hematoxylin and Eosin staining of the same 15-week-old thymus section showing medulla and cortex areas.

We next used immunofluorescence to assess whether monoclonal IgM secreted by immortalized thymic B cells and specific to adducts also reacted to thymic tissue. As shown in **Figure 6A**, IgM mAb representative for each of the anti-adduct reactivity type 1 to 3, positively stained thymic tissue. Control staining with fluorochrome-conjugated secondary antibody alone are presented in **Supplementary Figure 8**. **Figure 6B** depicts the Hematoxylin and Eosin staining of the same thymic specimen showing the corresponding medullary and cortical areas. Remarkably, reactivity was confined to the medulla with little to no visible staining of the thymic cortex. At higher magnification, the reactivity pattern revealed binding to specific medullary cells for the 3 types of anti-adduct antibodies.

## Discussion

IgM B cells compose the majority of medullary B cells in the human (3). Yet, unlike their mouse counterparts, these cells remain poorly characterized. Here, we investigated the reactivity at the clonal level of over 100 IgM B cells derived from thymus specimens collected from an infant, an adolescent, and an adult donor. Our study revealed that approximately 30-40% of thymic IgM B cell clones reacted to generic apoptotic cells, irrespective of the donor age. This frequency is much higher than that of peripheral blood IgM B cells sharing the same profile reactivity (2-5%). This significant enrichment of clones reactive to apoptotic cells in the human thymic compartment is a novel and intriguing finding likely related to their function in this organ. Further, through immunofluorescence staining, we show that these cells react selectively to the thymic medulla and more specifically to a subset of cells that still need to be identified.

We used a uniquely designed multi-dimensional ELISA panel composed of 60 PTM and other common chemical adducts to further identify the determinants recognized by thymic IgM B cells on apoptotic cells. We have previously utilized this platform to explore the anti-adduct humoral repertoire in human newborns and across the lifespan (12). In the present study, we observed a remarkable correspondence between reactivity to apoptotic cells and binding to a handful of specific adducts. Only a minority of the anti-apoptotic mAbs could not be further characterized with our ELISA, supporting the view that our panel covers the most relevant determinants. It is striking that a significant fraction of anti-apoptotic IgM cells could be assigned to only 4 different types based on their reactivity profiles. While not a formal demonstration, these results suggest that recognition of these adducts on apoptotic cells explain their overall reactivity. The predominant type corresponded to IgM reacting to the canonical oxidized epitope MDA. Anti-MDA antibodies were first described more than 30 years ago (18) and have been implicated in atherosclerosis and autoimmune diseases (19, 20). In addition to anti-MDA IgM, 3 other types were identified. Four clones displayed a typical polyreactive pattern in that they strongly reacted to a wide range of chemical adducts included in our panel. Their reactivity profile could not be further refined. Another type of clone reacted exclusively to NEDD8. NEDD8 is a ubiquitin-like protein involved in the neddylation process, which regulates cell growth, apoptosis, and embryogenesis through the activation of cullin-RING E3 ubiquitin ligases (21, 22). Lastly, several clones reacted to related derivatives of cysteine residue, including N-homocysteinyl-lysine, S-homocysteinyl-cysteine, and S-palmitoylated-cysteine. Homocysteinylation is a post-translational modification linked to oxidative stress and hyperhomocysteinemia, a risk factor for cardiovascular disease, stroke, and neurodegenerative conditions (23, 24). As these adducts are all related to oxidative stress and cell death processes, this could be another indication that they may facilitate the binding to apoptotic cells, potentially contributing to their clearance.

We recently reported on the highly restricted anti-adduct IgM immune repertoire in human newborns and infants (12). Until approximately 6 months of age, circulating IgM in cord blood and peripheral blood only react to a handful of adducts. Most significantly, among the 4 most primary targets of this early-life natural immunity are MDA, N-homocysteinyl-lysine and NEDD8, the 3 adducts recognized by IgM B cells derived from a 5-week-old thymus. Both observations are consistent and underscore the limited range of IgM B cell immunity following birth. Our findings also highlight the immunogenicity of the identified adducts among other ubiquitous chemical groups exposed on modified macromolecules.

Immunofluorescence staining of Hep-2 cells is a widely used method for the identification of antinuclear antibodies (ANAs) in the sera of patients with suspected autoimmune diseases, such as systemic lupus erythematosus or Sjörgens`s syndrome (16, 25). Different staining patterns, defined by the subcellular localization of the target antigens, can provide insights into specific autoimmune conditions (15, 16). Our results revealed that most antibodies reactive to apoptotic cells and adducts also recognized intracellular or intracellular antigens in Hep-2 cells, with some noticeable differences among the reactivity patterns. Unequivocal polyreactive clones (Type 4) were the most reactive to Hep-2, whereas those recognizing NEDD8 (Type 1) were the least reactive.

NAbs are an essential component of the immune system, providing rapid and broad-spectrum first-line protection against pathogens (26–28). NAbs also have crucial homeostatic functions associated with their capacity to bind self-determinants (29–31). For instance, NAbs specific to canonical oxidation epitope regulate thrombosis (32). Likewise, NAbs reactive to apoptotic cells promote efferocytosis and clearance of cellular debris. Another characteristic of NAbs is their polyreactivity, referring to the ability to bind unrelated antigenic structures, often including determinants on apoptotic cells (33). In this study, we observed an association between reactivity to apoptotic cells and to specific adducts, supporting the idea proposed in our previous report that recognition of particular chemical moieties exposed on multiple proteins or macromolecules could explain a clone’s apparent polyreactive pattern (12). Only four antibodies exhibited a pattern consistent with true polyreactivity, by reacting to a majority of chemical adducts in our panel. As suggested by Notkins and colleagues, such polyreactivity could be attributed to the flexibility of the antigen-binding sites (34).

In mice, thymic B cell autoreactivity was linked to their capacity to uptake and present self-antigens to single-positive thymocytes and delete reactive clones. As previously described, human thymic B cells also recognize generic autoantigens, which suggested that they exert a similar function (35). Our present study show that human thymic IgM B cells bind apoptotic cells presumably through the recognition of specific adducts further supporting their role in central tolerance mechanisms. This reactivity could not only facilitate the clearance of apoptotic cells or bodies but also promote the capture of large payloads of self-antigens within these cell remnants. This process might be particularly relevant in the thymus due to the increased level of apoptosis following negative and positive selection of T cells (36). This hypothesis is further supported by the selective staining of medullary cells by IgM mAb derived from thymic B cells. Considering that the thymic medulla is essentially a graveyard of negatively selected thymocytes (37, 38), it is plausible that these dying cells provide a constant source of autoantigens to resident B cells.

There are several inherent limitations to our study. First, due to the laborious type of work required to generate immortalized B cell clones, we only examined 3 subjects. Second, the number of clones derived from each specimen was also limited. However, besides these limitations, we were able to provide here the analysis of a representative sample of human thymic B cells of different ages. Our experiments revealed a high proportion of clones reactive to apoptotic cells among thymic IgM B cells irrespective of the age. Moreover, we provide here the first mapping of the anti-adduct IgM repertoire of human thymic IgM B cells and identified four distinct reactivity profiles. This finding provides a foundation for further investigations into the functional role of autoreactive thymic B cells.

## Data Availability

The original contributions presented in the study are included in the article/**Supplementary Material**. Further inquiries can be directed to the corresponding author.
