# Supplementary material for: Clones reactive to apoptotic cells and specific chemical adducts are prevalent among human thymic B cells
Source: Front Immunol. 2024 Oct 21;15:1462126. doi: 10.3389/fimmu.2024.1462126 (PMC11532181; doi:10.3389/fimmu.2024.1462126)
Supplement: Supplementary file 2 [file DataSheet1.pdf]

A

Thymus	Age	Total number of clones	IgM	IgG	IgA	Not determined
A	5 weeks	88	77	2	1	8
B	15 years	41	10	27	4	-
C	39 years	48	33	11	3	1

B

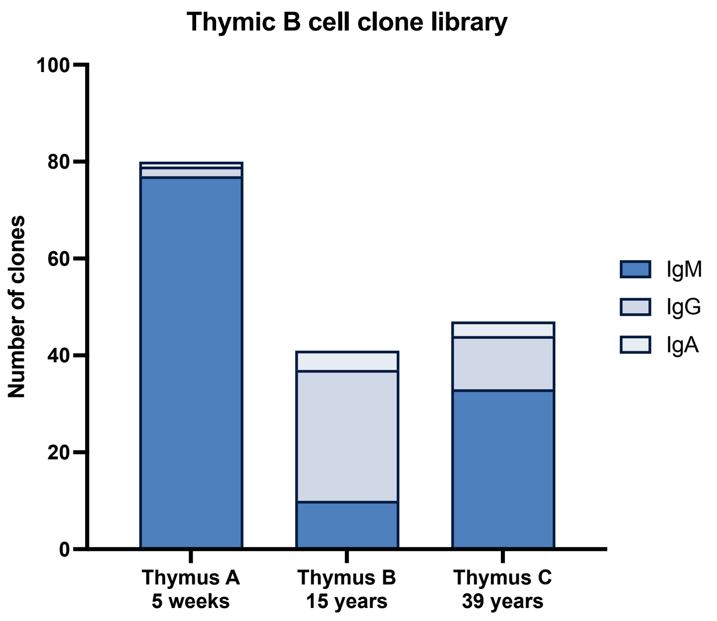

Figure S1. Distribution of different immunoglobulin class-secreting B cells among thymic B cells. (A) Total numbers of clones secreting IgM, IgG and IgA among immortalized thymic B cells (Thy A=5weeks, Thy B=15 years, Thy C=39 years) . (B) Bar graphs representation of the distribution of the different immunoglobulin class-secreting clones among thymic B cells.

A

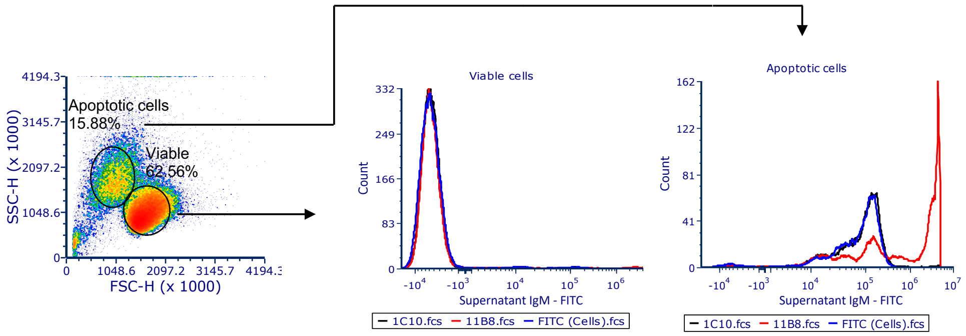

Apoptotic cells
15.88%
Viable
62.56%
SSC-H (x 1000)
FSC-H (x 1000)
4194.3
3145.7
2097.2
1048.6
0
Viable cells
Count
332
249
166
83
0
Supernatant IgM - FITC
1C10.fcs
11B8.fcs
FITC (Cells).fcs
Apoptotic cells
162
122
81
41
0
Supernatant IgM - FITC
1C10.fcs
11B8.fcs
FITC (Cells).fcs

B

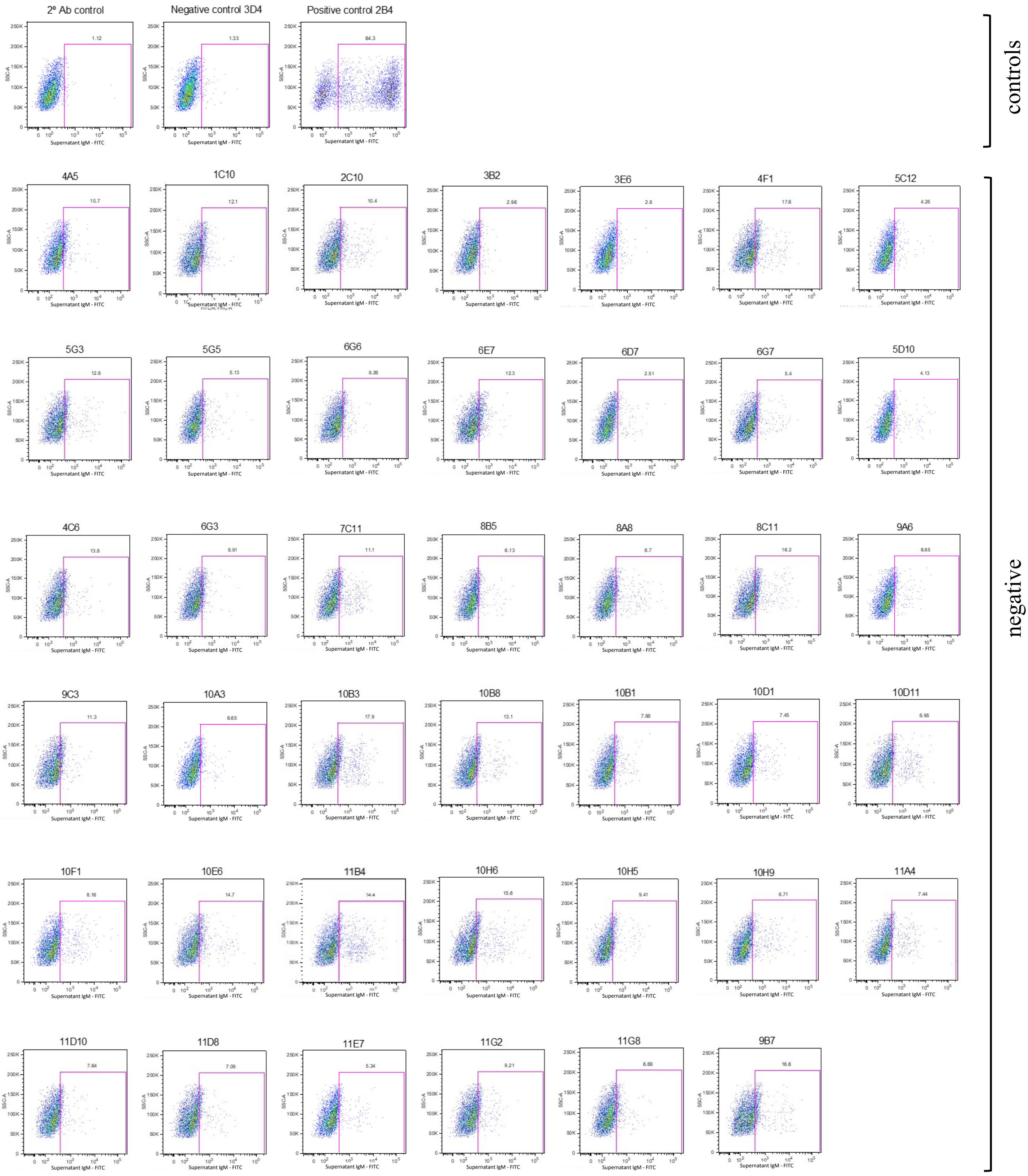
2° Ab control
1.12
Negative control 3D4
1.33
Positive control 2B4
64.3
controls
4A5
10.7
1C10
12.1
2C10
10.4
3B2
2.96
3E6
2.8
4F1
17.6
5C12
4.25
5G3
12.8
5G5
5.13
6G6
8.26
6E7
12.3
6D7
2.51
6G7
5.4
5D10
4.13
4C6
13.8
6G3
8.91
7C11
11.1
8B5
6.13
8A8
6.7
8C11
16.2
9A6
6.85
9C3
11.3
10A3
6.65
10B3
17.9
10B8
13.1
10B1
7.88
10D1
7.45
10D11
8.95
10F1
8.16
10E6
14.7
11B4
14.4
10H6
15.6
10H5
9.41
10H9
6.71
11A4
7.44
11D10
7.64
11D8
7.09
11E7
5.34
11G2
9.21
11G8
6.66
9B7
16.6
negative
SSC-A
Supernatant IgM - FITC

Thy A

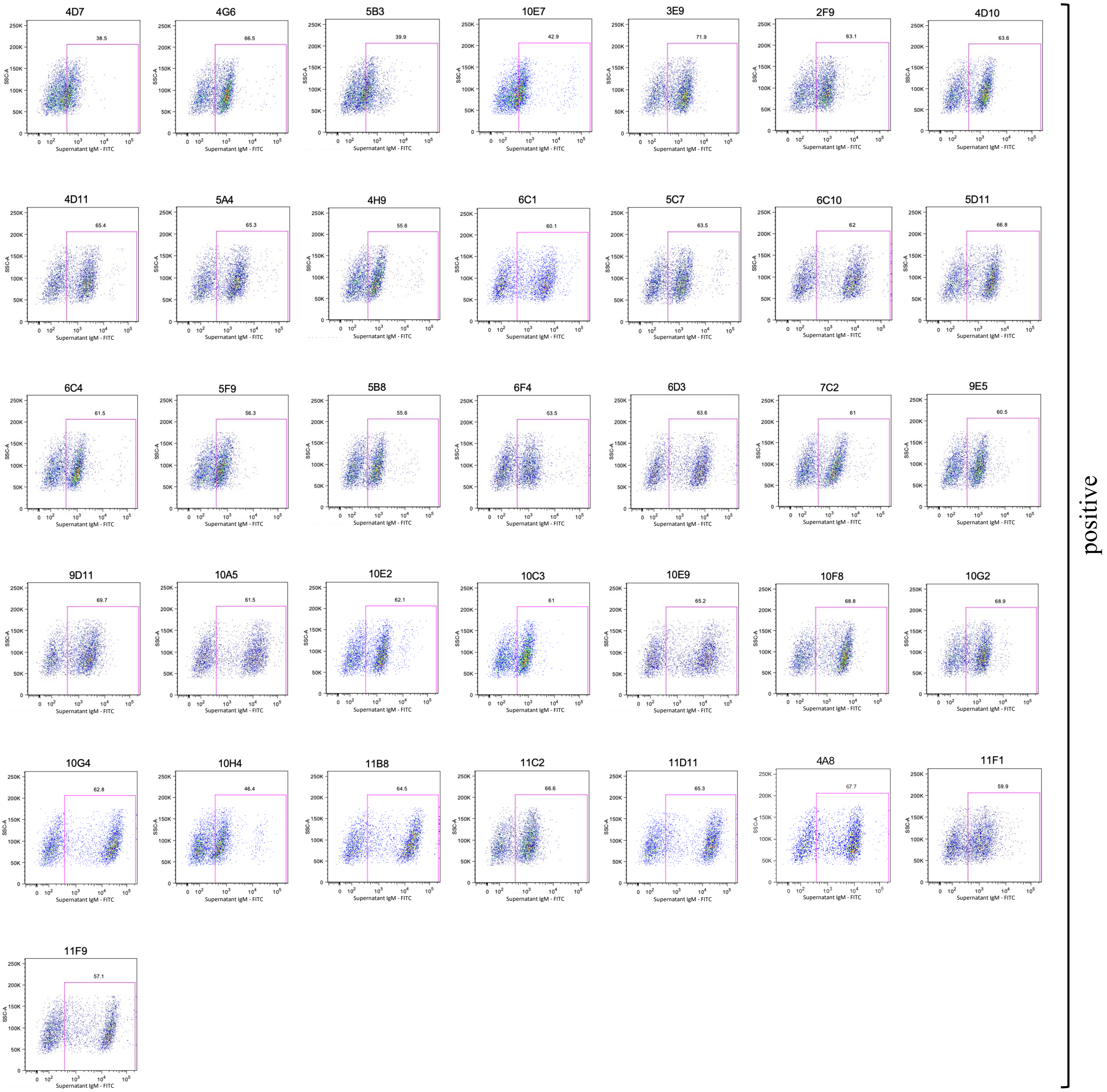
4D7
38.5
4G6
66.5
5B3
39.9
10E7
42.9
3E9
71.9
2F9
63.1
4D10
63.6
4D11
65.4
5A4
65.3
4H9
55.6
6C1
60.1
5C7
63.5
6C10
62
5D11
66.8
6C4
61.5
5F9
56.3
5B8
55.6
6F4
53.5
6D3
63.6
7C2
61
9E5
60.5
9D11
69.7
10A5
61.5
10E2
62.1
10C3
61
10E9
65.2
10F8
68.8
10G2
68.9
10G4
62.8
10H4
46.4
11B8
64.5
11C2
66.6
11D11
65.3
4A8
67.7
11F1
59.9
11F9
57.1
SSC-A
Supernatant IgM - FITC
positive

Thy A

C

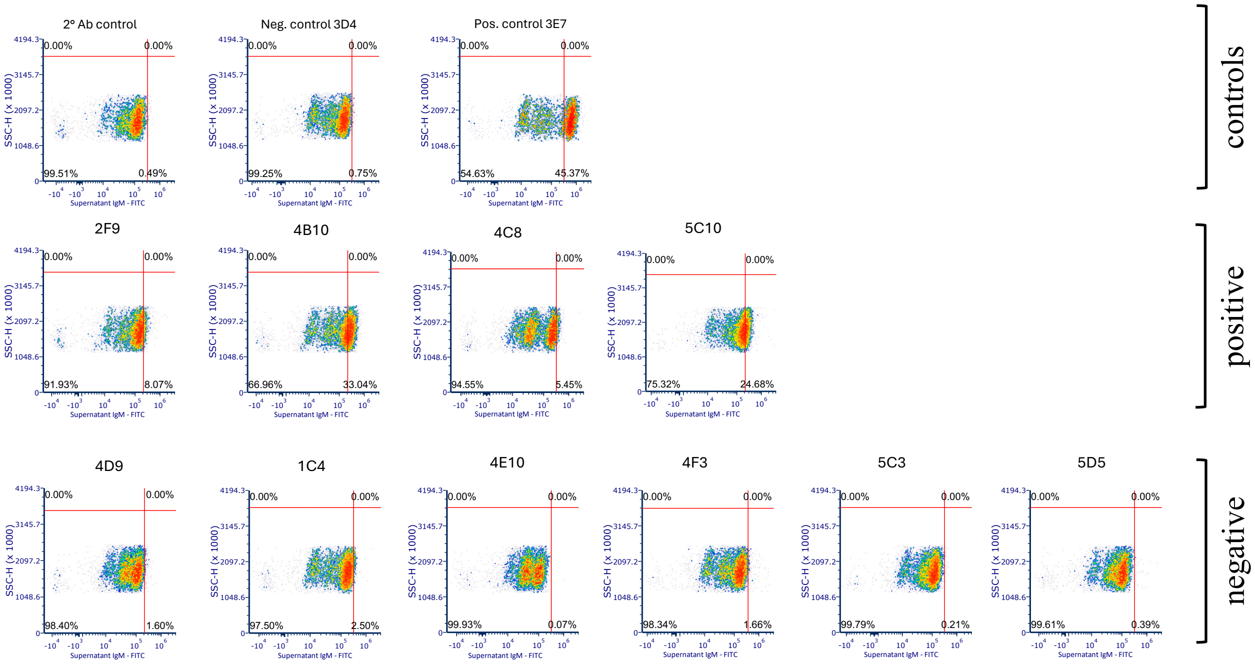

2° Ab control
Neg. control 3D4
Pos. control 3E7
controls
2F9
4B10
4C8
5C10
positive
4D9
1C4
4E10
4F3
5C3
5D5
negative
SSC-H (x 1000)
Supernatant IgM - FITC
99.51%
0.49%
99.25%
0.75%
54.63%
45.37%
91.93%
8.07%
66.96%
33.04%
94.55%
5.45%
75.32%
24.68%
98.40%
1.60%
97.50%
2.50%
99.93%
0.07%
98.34%
1.66%
99.79%
0.21%
99.61%
0.39%

Thy B

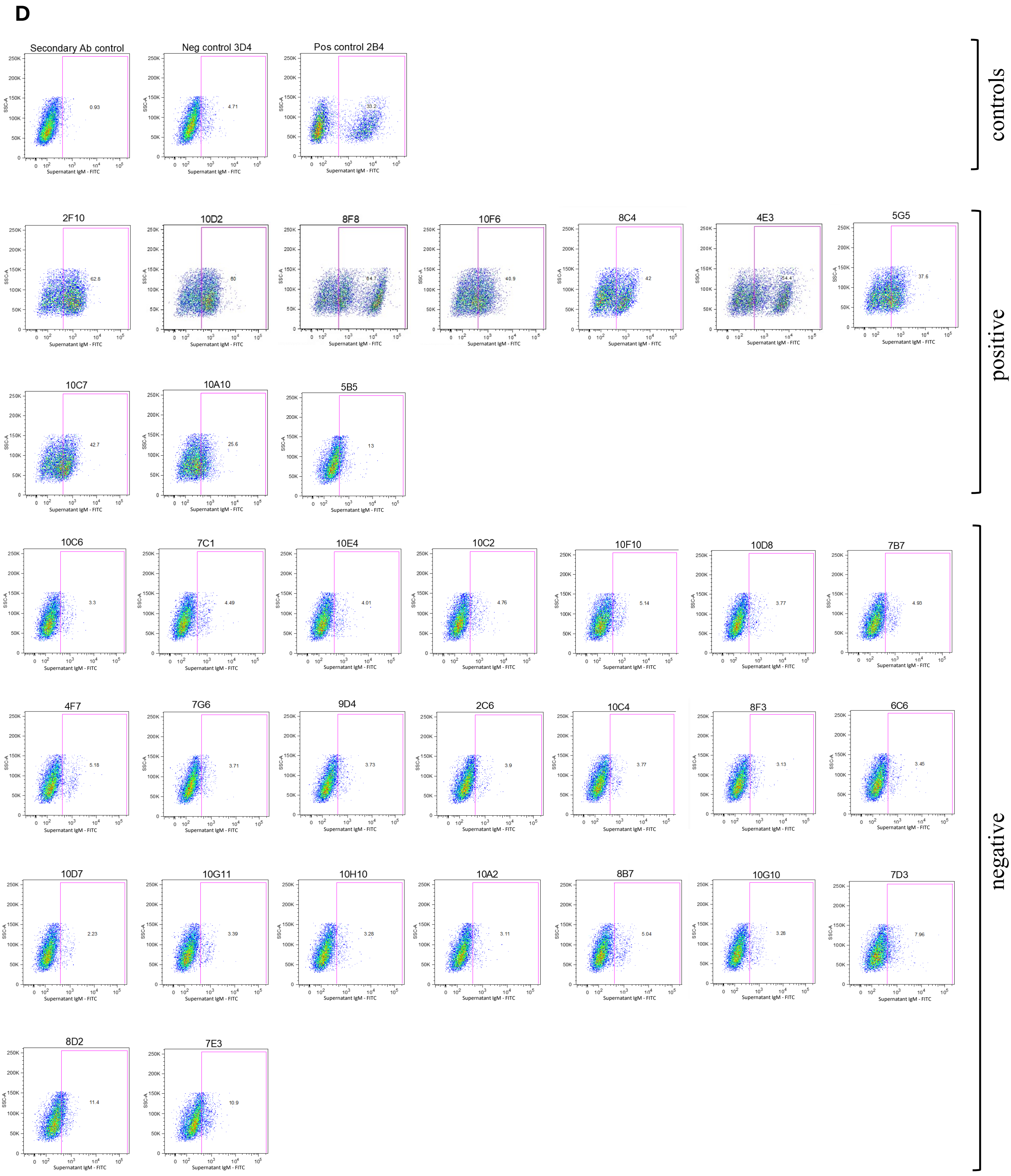

Thy C

Figure S2 Reactivity to apoptotic Jurkat T-cells cells of thymic IgM B cell clone. (A) Representative plots and histograms showing staining of a non-reactive clone (1C10, black line) and a reactive clone (11B8, red line) to apoptotic and viable Jurkat cells. Reactivity due to secondary antibody is also shown (blue line). (B-D) Reactivity to apoptotic Jurkat cells is shown for IgM B cell clones generated from a 5-week-old (B, Thy A), a 15-year-old (C, Thy B) and a 39-year-old thymus (D, Thy C).

	Donor 1	Donor 2	Donor 3	Donor 4
**Number of IgM B cell clones generated**	**469**	**361**	**220**	**240**
**Number of clones reactive to apoptotic cells**	**15**	**21**	**5**	**14**
**Frequency of clones reactive to apoptotic cells (%)**	**3.2**	**5.81**	**2.27**	**5.83**

Figure S3. Frequency of apoptotic cell-reactive clones among IgM B cells immortalized from peripheral blood of 4 healthy donors.

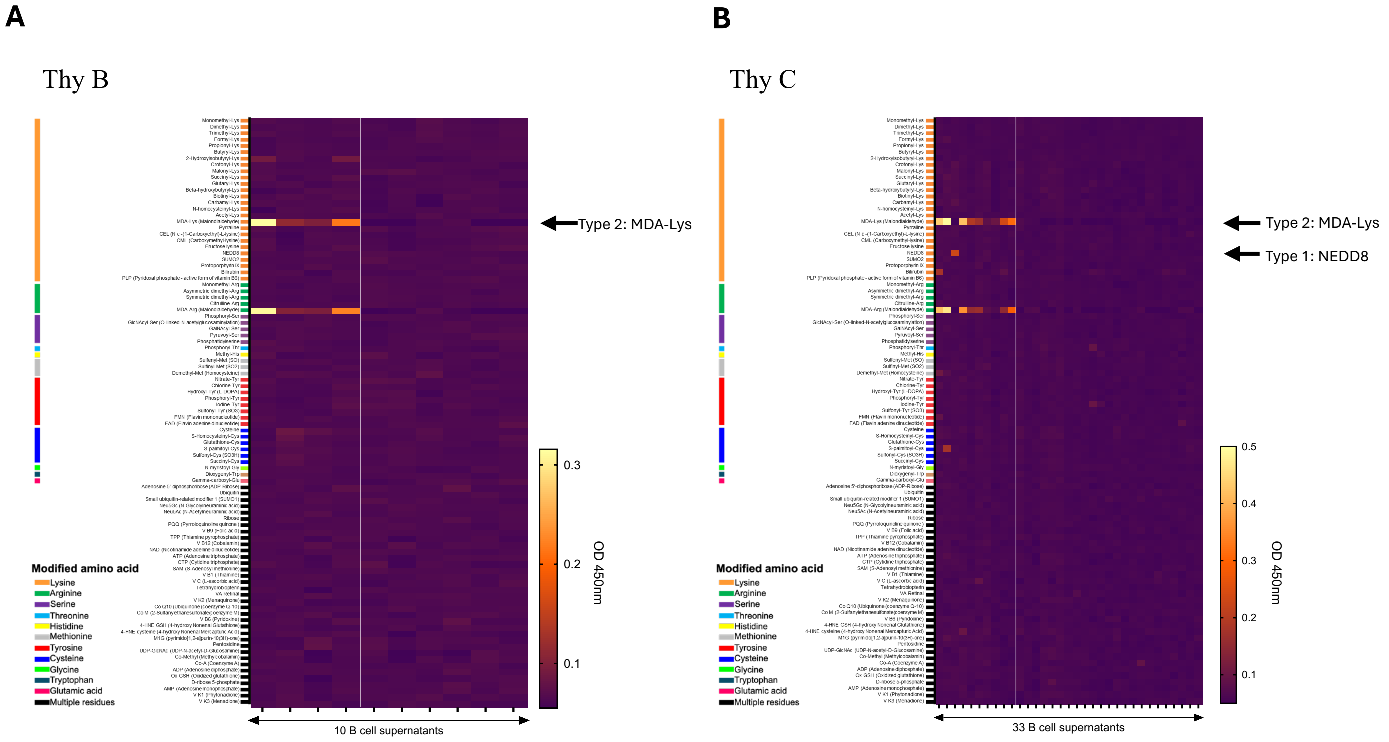

Figure S4. Reactivity of thymic IgM B cell clones generated from a 15-year-old thymus (A, Thy B) and a 39-year-oldthymus (B, Thy C) to 93 chemical adducts. B cell clones were categorized as reactive (left) or nonreactive (right).

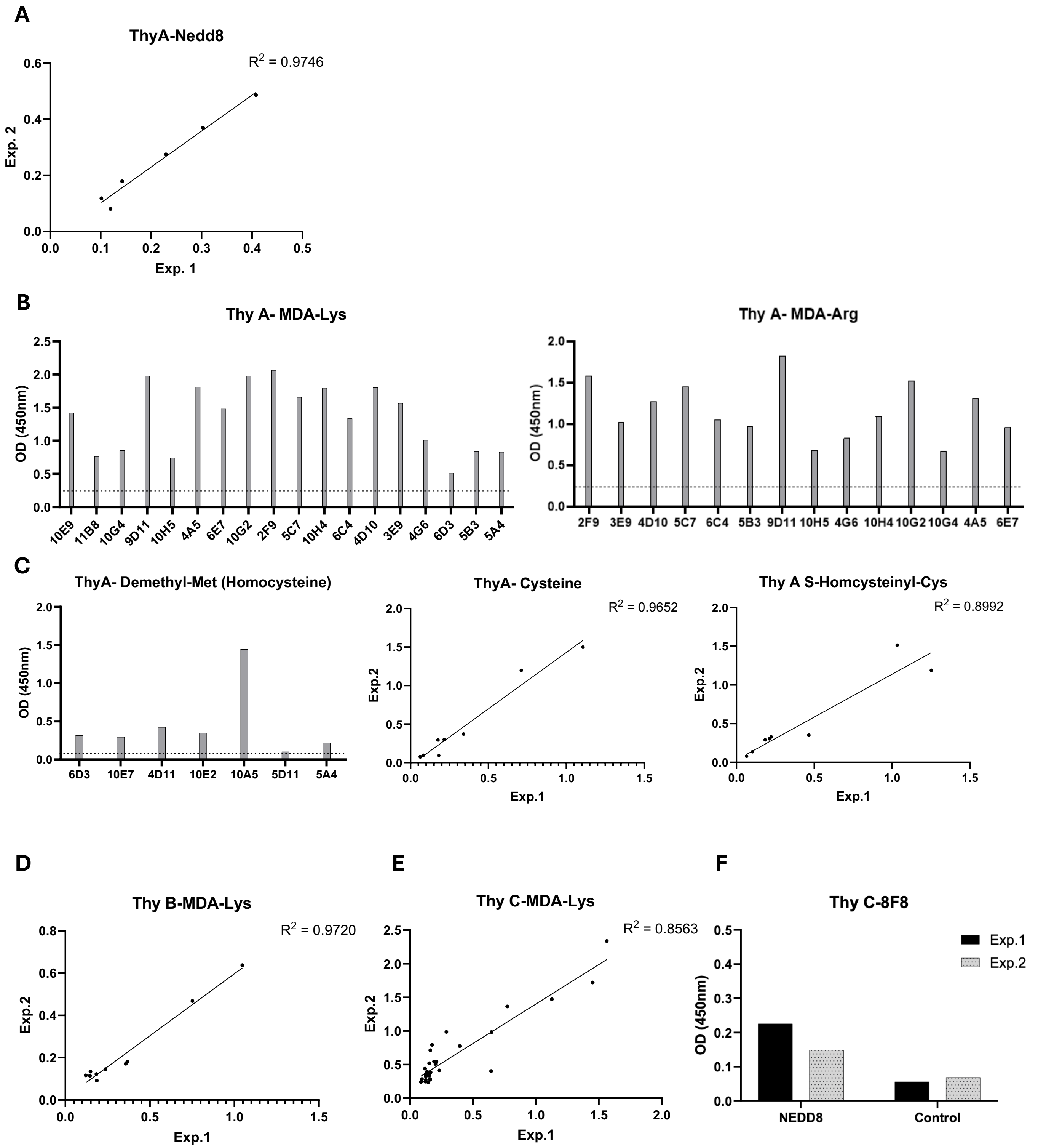

Figure S5. Validation of reactivity to chemical adducts by ELISA

Results are shown (i) as Linear regression of two individual separate experiments conducted in duplicates with the R squared value displayed, (ii) as bar graph showing the results of the second experiment including a dotted line representing the threshold based on the average OD from a negative control; and (iii) as comparative bar graphs for repeated experiments for: (A) Thy A samples towards NEDD8, (B) Thy A samples towards MDA-Lysine and MDA-Arg, (C) Thy A samples towards Demethyl-Methionine, Cysteine and Homocysteinyl-Cysteine, (D) Thy B samples towards MDA-Lysine (E) Thy C samples towards MDA-Lysine and (F) Thy C sample 8F8 towards NEDD8.

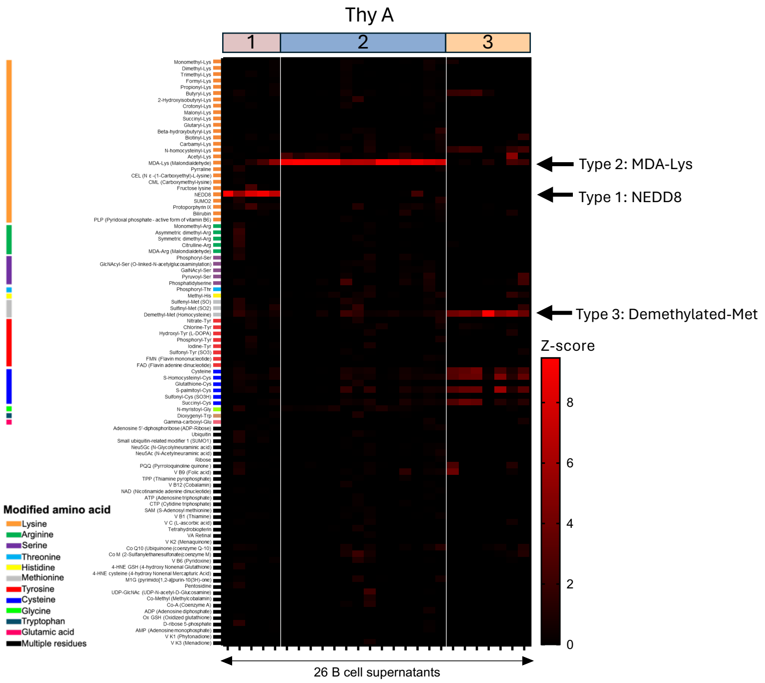

Figure S6. Characterization of thymic IgM B cell clone reactivity profiles.
Heatmap representation of the three different reactivity profiles: Type 1, reactivity to NEDD8, Type 2 reactivity to MDA-Lysine, and Type 3, dominant reactivity to demethylated-methionine (Homocysteine) for Thy A. Data were normalized and shown as Z-Score.

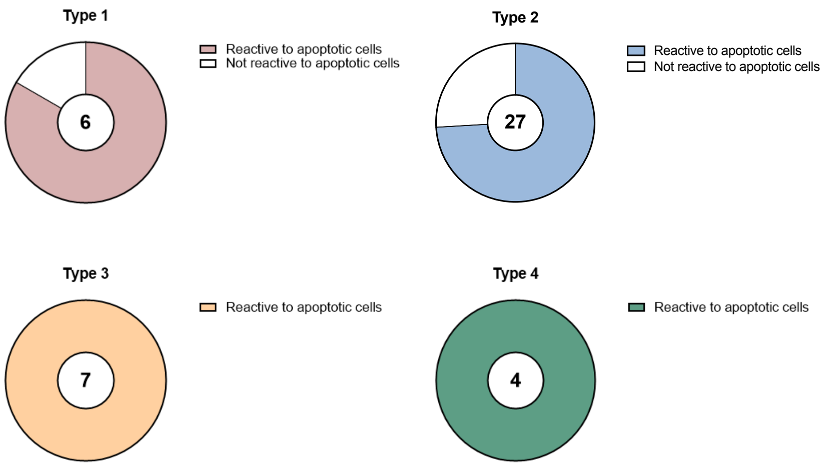

Figure S7. Number of clones reactive to apoptotic cells among different types of adduct-reactive IgM thymic B cells

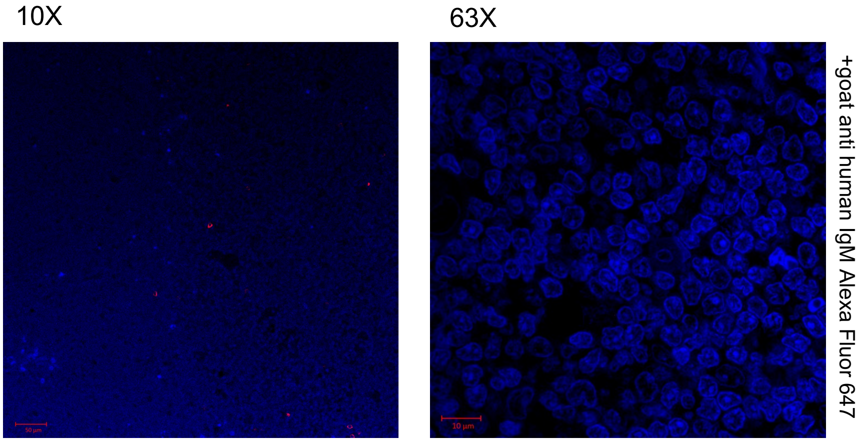

Figure S8. Immunofluorescence staining control. Sections of thymic tissue (5-week-old donor, Thy A) were stained with goat anti-human IgM secondary antibody alone.
